# Pathway linking health information behaviors to mental health condition during the COVID-19 infodemic: A moderated mediation analysis

**DOI:** 10.3389/fpubh.2022.924331

**Published:** 2022-08-29

**Authors:** Thomas Hongjie Zhang, Jen Sern Tham, Moniza Waheed, Jeong-Nam Kim, Jae-Seon Jeong, Peng Kee Chang, Abdul Mua'ti@Zamri Ahmad

**Affiliations:** ^1^Department of Communication, Universiti Putra Malaysia, Seri Kembangan, Selangor, Malaysia; ^2^Gaylord College of Journalism and Mass Communication, University of Oklahoma, Norman, OK, United States; ^3^Ulsan National Institute of Science and Technology, Ulsan, South Korea; ^4^School of Media and Communication, Taylor's University, Subang Jaya, Malaysia

**Keywords:** COVID-19 infodemic, information overload, health information behaviors, risk perception, mental health condition, Malaysia

## Abstract

**Background:**

The COVID-19 outbreak is no longer a pure epidemiological concern but a true digital infodemic. Numerous conflicting information and misinformation occupy online platforms and specifically social media. While we have lived in an infodemic environment for more than 2 years, we are more prone to feel overwhelmed by the information and suffer from long-term mental health problems. However, limited research has concentrated on the cause of these threats, particularly in terms of information processing and the context of infodemic.

**Objective:**

This study proposed and tested moderated mediation pathways from two types of health information behaviors (social media engagement and interpersonal communication) on information overload and mental health symptoms—long-term stress.

**Methods:**

We conducted a cross-sectional online survey between May and June of 2021 among the Malaysian public. The final sample size was 676 (*N* = 676). A conceptual model was built to guide the data analysis. We conducted structural equation modeling (SEM), moderation and mediation analyses to examine each direct pathway, moderating and mediating effects.

**Results:**

According to the pathway analysis, we found that, during the infodemic period, engaging COVID-19 information on social media positively associated with information overload, but interpersonal communication was negatively related to it. As the proximal outcome, there was also a positive association between information overload and the final outcome, perceived stress. The moderation analysis only reported one significant interaction: risk perception weakened the association between social media engagement and information overload. A conditional indirect effect was demonstrated and the indirect associated between social media engagement and perceived stress mediated through information overload was further moderated by COVID-19 risk perception.

**Conclusion:**

This research offers new grounds for understanding health information behaviors and their consequences in the COVID-19 infodemic. We particularly highlighted the distinct functions of health information behaviors in causing information overload, as well as the importance of personal health belief in this process. Our proposed model contributes to the strategies of developing health messaging strategies that may be utilized by public health researchers and health educators in the future.

## Introduction

It has been more than 2 years since the Coronavirus disease (hereafter COVID-19) firstly detected in Wuhan, a metropolitan city in central China, in December 2019. Since then, information channels, such as mass media, social media and interpersonal communication, have been instrumental in informing the public about the up-to-date situation of COVID-19, enhancing their knowledge, awareness, and prompting their preventive intentions toward the disease (1, 2). Technological changes on health information delivery systems such as social media are capable of disseminating health messages instantly during this time. For instance, one recent study found that consuming COVID-19 information on WeChat, Weibo, and TikTok mobilizes the Chinese citizens' intention and practice on precautionary measures (3, 4). In the US, information behaviors on social media also help the public develops the intention of wearing a facemask in public places (5).

Despite the documentation on the benefits of social media use during the pandemic, its utilization can create new problems. In response, the World Health Organization (WHO) has indicated that COVID-19 is accompanied by a true social media infodemic, as information and misinformation about the disease spreads online (6). The phenomenon of infodemic is evident in the online environment, where misperceptions toward the virus, politicalized contents regarding preventive measures, conspiracy theories, and manipulated anti-vaccination messages are widely spread without censors (7, 8). Therefore, examining relevant information behaviors and consequences in the context of infodemic is of paramount importance.

### Study rationale and hypotheses development

When infodemiological consideration becomes a severe side effect of the COVID-19 pandemic, many related problems emerged (7, 9). One prominent issue attracting scholarly attention is information overload (IO) (10), a situation where individuals feel overwhelmed and confused about a specific health topic after being inundated with an informational mixture containing verified and unverified health information from various sources (11). Previous studies have revealed that IO is one of the negative consequences of health information engagement (12). It occurs when individuals fail to process newly obtained health information as the information environment is full of confused, heterogeneous, and misleading contents (13). If individuals suffer from IO, as a result, their knowledge acquisition, quality of life, and mental health are very likely to be affected (11, 14, 15).

The conceptualization and theorization of IO in public health remains unclear despite ample studies examining its role in studies on cancer (11, 16), nutrition (17), and the COVID-19 pandemic (18, 19). First, previous studies fail to include IO as a limitation of message processors in the information process (19). These studies only examined the relationship between IO and demographical and psychosocial factors, such as family cancer history, anxiety, and sadness (11, 17). Meanwhile, the operational definition of IO in existing studies is equivocal. Some studies considered IO as a result of media usage (20), whilst others recognized IO as an existing “environmental stimulus,” thus linking IO with psychological reactions or an immediate consequence (e.g., information avoidance) (10, 19, 21). Hence, ambiguities concerning the concept's content and boundaries as well as measurement problems limit cumulative theory building and easy adaption in health communication. In this study, we proposed that the understanding of IO should adhere to the most forthright reasoning in information processing: Someone may suffer from IO after engaging the relevant media content (12) rather than presuming he or she is immersed in an overwhelmed information environment. Consequently, we focus on the information engagement on social media, while linking social media engagement to IO as a proximal outcome and perceived stress as one prominent long-term mental health condition during the COVID-19 infodemic, as the final outcome (22).

We also included interpersonal communication as another information-gathering strategy for acquiring health information, as it is defined as a critical information behavior during a pandemic (2, 23). In this study, interpersonal communication refers to the real-time and face-to-face discussions for obtaining COVID-19 health information. During the time of the infodemic, individuals already have too much conflicting informational input during their daily social media usage. When they communicate COVID-19 issues with their family, friends and other social networks, their likelihood of experiencing IO would be higher since the information obtained from interpersonal networks is seemingly conflicting. The trustworthiness and credibility of obtained information somehow cannot be ensured. For example, a study in South Korea during the earlier stages of the outbreak reported that communicating COVID-19 topics with family, friends and co-workers positively triggered the likelihood of IO (20). Therefore, we propose the following hypotheses:

*H1: Social media engagement is positively associated with information overload*.

*H2: Interpersonal communication is positively associated with information overload*.

Regardless of how previous studies defined IO, the majority demonstrated that IO is associated with immediate responses, such as information anxiety (24) and intentions to reject further information (10). However, they ignored the predictive power of IO on long-term psychological or health outcomes. As the impact of health information acquisitions on long-term mental health symptoms becomes a critical concern during the COVID-19 outbreak (25), it is reasonable to examine the patterns of individuals engaging in health information and the implications in an infodemic environment. Especially, since this infodemic has persisted for over 2 years, individuals feel overwhelmed with the wealth of information on COVID-19 surrounding them, causing stress and contributing to pandemic fatigue. A recent study found that stress-related contents were more likely to be expressed than worry- and fear-related ones after April 2020 on the COVID-19 Twitter posting trend (22). Therefore, governments and medical authorities have begun to educate the public on preventative measures, publish scientific reports, as well as plan and implement vaccination programs that can serve as uncertainty reducer for individuals (26). They were less likely to feel worry and fear, but more likely to be stressed in the long run. Considering this, we include stress as a health outcome and hope to learn how information processing during the infodemic contributes to this mental health condition. Since IO is caused by information consumption and predicts several other outcomes (12, 27), it is concomitantly essential to consider IO as a mediator between information behaviors and health outcomes. Therefore, the following hypotheses are proposed:

*H3: Information overload is positively associated with perceived stress*.

*H4: Information overload positively mediates the association between (a) social media engagement, (b) interpersonal communication, and perceived stress*.

Furthermore, apart from understanding health IO and subsequent mental health outcome in the COVID-19 infodemic through the linear fashion, the mechanisms of mediated communication in public health settings are much more complicated and dynamic. From the theoretical aspect, the ecological model of communication (28) depicts that the process of public health communication or health-related media usage involves influences from various contextual factors including personal, interpersonal, organizational, and societal or cultural levels. Interplays between these contextual factors and communicative actions could jointly affect health outcomes (29). The contextual factors are broad, complex, and multidimensional (30). Therefore, it is beyond the objective of this study to comprehensively review moderators on the pathways between information behaviors and IO. We select risk perception as an example of contextual influences, as many empirical studies in different public health contexts accentuated that personal health beliefs, especially perceived likelihood, severity, seriousness, and susceptibility regarding a health threat (all under the terminological umbrella of “risk perception”), are predominant psychological factors that affect individuals' preventive intentions and coping behaviors, including health information acquisition (31, 32). Although considering risk perception as a contextual factor in the COVID-19 infodemic context is rare, especially its influence on the process of causing IO, logical reasoning facilitates our arguments. Risk belief related to the COVID-19 pandemic can impact health information processing, either by strengthening or weakening the association between information behaviors and the outcomes. Individuals who believe their chances of getting COVID-19 and those around them are high may have a better awareness of the pandemic and are more familiar with the most recent information on preventive measures than those who believe they are not at danger. In the same vein, high perceived risk individuals are keener to use the information they have obtained and accessible health services to evaluate the current situation, make health decisions, and take preventive measures. It is reasonable to say that individuals with sufficient risk perceptions are less likely to feel overwhelmed, fatigued, and experience other adverse outcomes than their counterparts after frequently consuming COVID-19 information from different channels. This proposition echoes Street's (28) ecological model regarding the moderating role of self-related health concepts, such as attitudes and beliefs. Besides, it is also worth noting that our study is not the first to offer this logical thought. For other health issues, a study focusing on the MERS outbreak in South Korea reported that risk perception strengthens the relationship between health information seeking and preventive behaviors practices (33). Regarding cancer issues, Zhuang and Guan (34) also found that risk perception moderates the association between previous cancer information seeking experiences and breast cancer screening among female Americans. As such, we propose the following:

*H5: COVID-19 risk perception negatively moderates the positive association between (a) social media engagement, (b) interpersonal communication, and information overload*.

Considering risk perception moderates the positive association between COVID-19 health information behaviors and IO, it is also feasible to propose that risk perception could conditionally bring effects to the indirect pathway from information behaviors (antecedents) to perceived stress (outcome) through IO (the mediator and proximal outcome). Therefore, we postulate:

*H6: COVID-19 risk perception negatively moderates the indirect effect of (a) social media engagement and (b) interpersonal communication on perceived stress through the mediating role of IO*.

**Figure 1:**
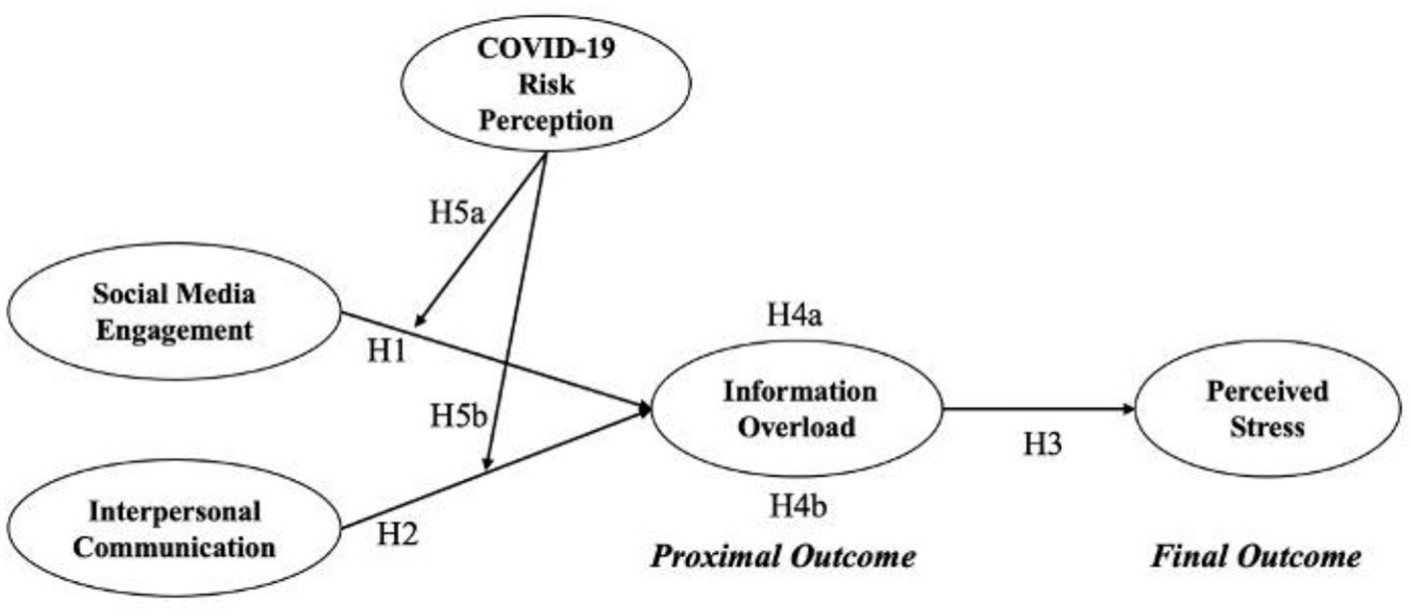
Conceptual model.

Taken all together, a pathway model is conceptualized (Figure 1).

## Methods

### Data collection

A cross-sectional survey was conducted from May to June 2021 in Malaysia after obtaining ethical approval from the authors' affiliated institution [UPM/TNCPI/RMC/JKEUPM/1.4.18.2 (JKEUPM)], when the country was under a full lockdown. We used a set of online questionnaires in three versions to recruit respondents (i.e., in English, Malay, and Chinese; the three main languages Malaysians use). Due to the safety measures announced by the government during the lockdown, we were unable to collect data through physical ways. Thus, we generated the survey items into Google Form and then distributed the links on authors' different social media platforms. Participants were recruited by distributing a one-page recruiting message to community leaders and social media influencers in the authors' Facebook, Twitter, Instagram, and WhatsApp groups. In the recruitment message, we included a brief introduction to the study's purpose, data collection procedures, the voluntary nature of participation, declarations of anonymity and confidentiality, and notes for filling out the questionnaire, as well as links to English, Malay and Chinese language versions of the online questionnaires.

All respondents were above 18 years old and therefore involved no minors. The study participants were given no incentive for their participation. Participants gave consent to willingly participate in the survey by clicking the “continue” button, which would direct them to complete the self-administered questionnaire. After employing convenience and snowball sampling methods concurrently, a total of 776 surveys were initiated. Only surveys that were missing <10% of data were retained (35). Hence, we included a total of 676 responses in the final analysis. The description of demographic information is shown in Table 1.

**Table 1:** Demographic information of the respondents (*N* = 676).

**Demographic factors**		***n***	**%**
**Gender**	Male	296	43.8%
	Female	380	56.2%
			
**Age**	Mean: 32.87, SD: 10.60		
			
**Ethnicity**	Malay	327	48.4%
	Chinese	269	39.8%
	Indian	28	4.1%
	Non-muslim bumiputra	52	7.7%
			
**Religion**	Buddhism	148	21.9%
	Christianity	135	20.0%
	Islam	334	49.4%
	Taoism & traditional Chinese beliefs	33	3.3%
	Hinduism	24	3.6%
	Non/Atheism	13	1.9%
			
**Education**	Primary school	1	0.1%
	Secondary school	45	6.7%
	High school	47	7.0%
	Diploma	122	18.0%
	Bachelor's degree	368	54.4%
	Postgraduate degree	93	13.8%
			
**Staying status**	Metropolitan area	308	45.6%
	Urban area	273	40.4%
	Rural area	95	14.1%

### Measurement

The survey questionnaire used in this study contained six sections, including measured variables in the conceptual model and demographic information. A total of 20 items were involved in measuring five constructs in the conceptual model. We used a 6-point scale to measure each item. The questionnaire in English can be accessed in Supplementary material.

#### Antecedent factors

Social media engagement, one of the health information behaviors, was measured by two items ranging from 1 (not at all) to 6 (very much) (36), which are “How often did you receive/express COVID-19 information on social media platforms (e.g., Facebook, Twitter, Instagram, WhatsApp, Telegram, WeChat) during the last 7 days?” To treat this as a continuous variable, all items were summed and averaged to create a composite score, with a higher score indicating a higher level of social media engagement (α = 0.85, *M* = 5.37, *SD* = 0.76).

Interpersonal communication as another health information behavior to obtain COVID-19 information was measured by four items (1 = not at all to 6 = very frequently), adopted from Ho et al. (2). Four interpersonal information sources were family members, friends, colleagues, and healthcare providers. We averaged the responses and created a composite score. Higher score indicates a higher frequency of discussing COVID-19 topics with interpersonal networks (α = 0.76, *M* = 4.42, *SD* = 0.96).

#### Proximal outcome factor

IO was served as the proximal outcome and mediator in our conceptual model. Its measurement included five items (ranging from 1 = strongly disagree to 6 = strongly agree) which was adopted from Costa et al.'s (37) simplified Cancer Information Overload Scale (13). We replaced “cancer” with “COVID-19” in the items. The responses were summed up and averaged, with higher score showing higher level of IO (α = 0.91, *M* = 5.44, *SD* = 0.66).

#### Final outcome factor

The final outcome variable in the conceptual model is the long-term mental health condition, perceived stress. It was measured with two items on a 6-point scale ranging from 1 (strongly disagree) to 6 (strongly agree) (38). These were (1) “Currently, I feel so down in the dumps that nothing could cheer me up” and (2) “Currently, I feel downhearted and blue.” All responses were summed and averaged to create a single index, with higher score indicating higher stress level (α = 0.84, *M* = 4.84, *SD* = 1.07).

#### Moderating factor

As a type of personal health belief, COVID-19 risk perception served as a moderator in the conceptual model. This instrumentation was guided by Dryhurst et al. (39). Seven items were included using a 6-point scale, ranging from 1 (strongly disagree) to 6 (strongly agree). These items included subdimensions under the concept of risk perception, such as perceived seriousness, perceived severity, perceived susceptibility, and comparative risk belief at the individual, societal and global levels. We summed and averaged these seven items to consider it as continuous, with a higher score representing higher risk perception (α = 0.93, *M* = 5.12, *SD* = 0.89).

### Data analysis

We performed two statistical methods to analyze the conceptual model, structural equation modeling (SEM), as well as moderation and mediation analysis in PROCESS macro. First, the pathway analysis was conducted using SEM through lavaan package in R. In the structural model, two types of information behaviors, social media engagement, and interpersonal communication, were considered exogenous variables, the proximal outcome, IO, and the final outcome perceived stress were endogenous variables. Demographic variables such as gender, age, ethnicity, religion, and education level were treated as control variables. We used maximum likelihood estimation to examine the pathway coefficients of the hypothesized model. To establish the proposed model and evaluate its fit, the following criteria were considered: (1) relative chi-square (*x*^2^/df), (2) comparative fit index (CFI), (3) Tucker–Lewis index (TLI), (4) root mean square error of approximation (RMSEA), and (5) standardized root mean square residual (SRMR). If the model has a good statistical fit with the data, the value of the relative chi-square should fall between 1.0 and 5.0, and the CFI and TLI values need to be higher than 0.95, RMSEA should be close to 0.06, and SRMR values should be less than 0.08 (40).

Additionally, we used the PROCESS macro in R to examine simple mediation (H4) and moderation (H5) in the conceptual model. This method is suitable for analyzing moderation and mediation relationships and generating moderated mediation effects in a predefined model (41). Two PROCESS models were used to analyze these relationships accordingly. First, model 4 was employed to assess the simple mediation effects of IO on the association between health information behaviors (i.e., social media engagement and interpersonal communication) and perceived stress. Second, we applied model 7 to examine the direction relationships and moderated mediation effects. We adopted Preacher et al.'s (42) normal theory-based approach to understand the conditional indirect effect (i.e., moderated mediation, H6). Moderator values at three levels were taken into account, including low (1 standard deviation below the mean), medium (mean), and high (1 standard deviation above the mean). Furthermore, to determine these statistical effects, we practiced bootstrapping method with 5,000 bootstrap samples at each stage of the analysis; 95% confidence interval (CI) served as a pivotal reference to determine the effect size and level of statistical confidence.

## Results

**Figure 2:**
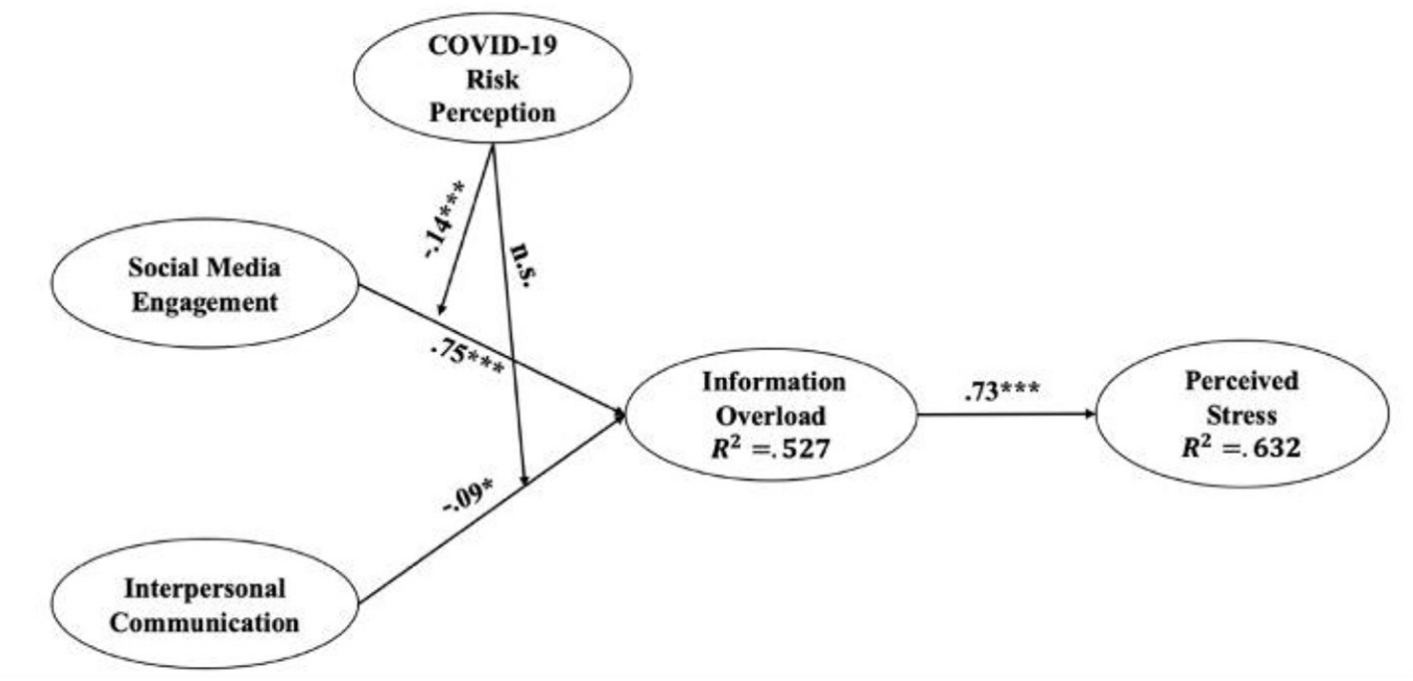
Conceptual model after analysis. *: *p* < 0.05, **: *p* < < 0.01, ***: *p* < 0.001.

The descriptive statistics for key variables, along with the confirmatory factor analysis (CFA) results, are demonstrated in Table 2. Pertaining to the pathway analysis by using SEM, our conceptual model showed a good fit: *x*^2^/df = 3.162, CFI = 0.966, TLI = 0.960, RMSEA = 0.057 [95% CI: (0.05, 0.06), *p* = 0.028], SRMR=0.036. This model explained 52.7% of the variance in the proximal outcome, IO (*R*^2^ = 0.527) and 63.2% in the final outcome, perceived stress (*R*^2^ = 0.632). Specifically, as shown in Figure 2, social media engagement was positively associated with IO (β = 0.75, *p* < 0.001), which supports H1. Interpersonal communication revealed a negative association with IO (β = −0.09, *p* = 0.010), which means H2 was not supported. Furthermore, the result showed that IO was positively associated with perceived stress (β = 0.73, *p* < 0.001), supporting H3.

**Table 2:** Descriptive statistics and confirmatory factor analysis (CFA) of measured variables.

**Item name**	***Mean***	***Median***	***SD***	**Factor loading**
*Social media engagement* (α = 0.85)	5.37	5.50	0.76	
SME1				0.79
SME2				0.71
*Interpersonal communication* (α = 0.76)	4.42	4.50	0.96	
IC1				0.70
IC2				0.82
IC3				0.75
IC4				0.46
*COVID-19 risk perception* (α = 0.93)	5.12	5.71	0.89	
RP1				0.86
RP2				0.88
RP3				0.83
RP4				0.84
RP5				0.82
RP6				0.80
RP7				0.66
*Information overload* (α = 0.91)	5.44	5.40	0.66	
IO1				0.82
IO2				0.84
IO3				0.80
IO4				0.84
IO5				0.83
*COVID-19 stress* (α = 0.84)	4.84	5.00	1.07	
Stress 1				0.84
Stress 2				0.87

Two statistical models were built to analyze moderation and meditation effects accordingly. Model A involves the analyses relating to the pathway from social media to perceived stress. First, regarding whether IO mediates the association between social media engagement and perceived stress. PROCESS macro model 4 was applied. The result (Table 3) showed a significant mediation effect [*Mediation Index* = 0.57, *SE* = 0.06, 95% CI = (0.46, 0.69)], supporting H4a. Particularly, social media engagement was positively associated with IO [*b* = 0.79, *SE* = 0.04, *t* = 17.63, 95% CI = (0.70, 0.88), *p* < 0.001], and increased level of IO was positively related to perceived stress [*b* = 0.71, *SE* = 0.04, *t* = 16.42, 95% CI = (0.63, 0.80), *p* < 0.001]. A partial mediation effect was thus generated.

**Table 3:** Moderation and mediation analysis results by using PROCESS macro.

**Pathways**	**b**	**SE**	**t**	**95% CI**	***P* value**
***Model A: Social media engagement***					
SEM → IO	0.79	0.04	17.63	0.70–0.88	**<0.001**
IO → Stress	0.71	0.04	16.42	0.63–0.80	**<0.001**
SEM → Stress	0.22	0.05	4.13	0.11–0.32	**<0.001**
Risk → IO	0.26	0.05	4.38	0.14–0.37	**<0.001**
Risk → Stress	0.26	0.06	4.39	0.14–0.38	**<0.001**
SEM*Risk → IO	**−0.14**	**0.03**	**−4.58**	**−0.21–** **−0.08**	**<0.001**
SME → IO → Stress	**0.57**	**0.06**	/	**0.46–0.69**	/
***Conditional indirect effect***					
Low COVID-19 risk perception (M-1SD)	0.48	0.06	9.17	0.35–0.60	/
Moderate COVID-19 risk perception (M)	0.41	0.06	7.91	0.28–0.54	/
High COVID-19 risk perception (M+1SD)	0.35	0.07	6.47	0.23–0.49	/
					
***Model B: Interpersonal communication***					
IC → IO	−0.09	0.03	−2.91	−0.13– −0.03	**0.003**
IO → Stress	0.82	0.03	24.72	0.76–0.89	**<0.001**
IC → Stress	0.06	0.03	2.00	−0.04–0.12	0.053
Risk → IO	0.30	0.04	3.82	0.10–0.47	**<0.001**
Risk → Stress	0.24	0.06	3.97	0.13–0.38	**<0.001**
IC*Risk → IO	−0.40	0.04	−1.47	−0.13–02	0.130
IC → IO → Stress	**0.10**	**0.03**	/	**0.04–0.16**	/

Unstandardized coefficient values (b) were reported, 5,000 bootstrap sample approach was applied to determine mediation and moderation effects, SEM, social media engagement; IC, interpersonal communication; IO, information overload; SE, standard error; 95% CI, 95% confidence interval; moderating relationships were demonstrated by “variable A*variable B,” gender, age, ethnicity, religion, and education level were included as covariates, Model B could not demonstrate a moderated mediation (conditional indirect) effect as the interaction between IV and moderator was not significant. Bold values refer to statistically significant pathways.

**Figure 3:**
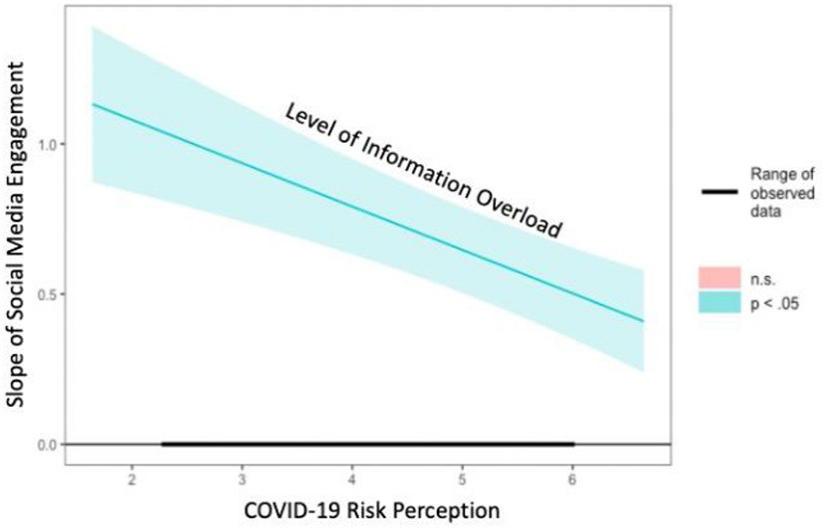
Johnson-Neyman plot for the interaction effect between risk perception and social media engagement on IO.

With regards to the moderation effect of COVID-19 risk perception in the relationship between social media engagement and IO, we used PROCESS macro model 7 to examine it. The result of moderation analysis (Table 3) indicated that there was a significant and negative two-way interaction between social media engagement and COVID-19 risk perception [*b* = −0.14, *SE* = 0.03, *t* = −4.58, 95% CI = (−0.21, −0.08), *p* < 0.001]. This means that risk perception weakened the association between social media engagement and IO, supporting H5a. The Johnson-Neyman plot (43) demonstrated that those with stronger COVID-19 risk perception are less likely to experience IO when they obtain more information from social media (Figure 3).

Furthermore, the results also demonstrated a significant moderated mediation effect (Table 3). The relationship between social media engagement and perceived stress mediated through IO was further moderated by COVID-19 risk perception [*Moderated Mediation Index* = −0.11, *Boot SE* = 0.02, 95% CI = (−0.14, −0.06)] which supports H6a. Specifically, the indirect effect of social media engagement on perceived stress was stronger among respondents with lower level of COVID-19 risk perception [*b* = 0.48, *Boot SE* = 0.06, 95% CI = (0.35, 0.60)], compared to respondents hold moderate level [*b* = 0.41, *Boot SE* = 0.06, 95% CI = (0.28, 0.54)] and higher level of COVID-19 risk perception [*b* = 0.35, *Boot SE* = 0.07, 95% CI = (0.23, 49)]. In other words, this result implies that those who hold a higher level of COVID-19 risk perception would be less likely to perceive stress even with the same degree of social media engagement mediated through IO.

Pertaining to the pathway from interpersonal communication to perceived stress, we formed Model B to analyze relevant mediation and moderation effects (Table 3). First, after analyzing data in PROCESS macro model 4, a significant mediation effect of IO in the association between interpersonal communication and perceived stress was found [*Mediation Index* = 0.10, *SE* = 0.03, 95% CI = (0.04, 0.16)], which supports H4b. Specifically, interpersonal communication was negative associated with IO [*b* = −0.09, *SE* = 0.03, *t* = −2.91, 95% CI = (−0.13, −0.03), *p* = 0.003], and decreased level of IO in turn positively related to perceived stress [*b* = 0.82, *SE* = 0.03, *t* = 24.72, 95% CI = (0.76, 0.89), *p* < 0.001]. Since we did not find a significant direct association between interpersonal communication and perceived stress [*b* = 0.06, *SE* = 0.03, *t* = 2.00, 95% CI = (−0.04, 0.12), *p* = 0.053], a full mediation effect was obtained. However, the results did not find a moderating effect of COVID-19 risk perception in the relationship between interpersonal communication and IO [*b* = −0.40, *SE* = 0.04, *t* = −1.47, 95% CI = (−0.13, 0.02), *p* = 0.130]. It was also unable to generate a moderated mediation effect. H5b and H6b failed to be supported.

## Discussion

This study uncovered the pathways of how health information behaviors cause IO on COVID-19 topics and perceived stress as one of the significant long-term mental health conditions in the COVID-19 context. We also included risk perception, a crucial personal health belief, as the moderator to analyze the interaction effects accordingly. Our model followed the most fundamental and simplified definition of IO, a consequence of engaging information from media channels (12). Since social media platforms have been recognized as the primary avenue where the public usually access health information in the era of infodemic (44), we thus proposed social media engagement as the information engagement approach (i.e., antecedent factor) in the conceptual model. Not surprisingly, the regression results reported that social media engagement was positively associated with IO, and the standardized coefficient was relatively high. It means, when someone engages more COVID-19 information during their daily social media usage, he or she is very likely to feel overwhelmed and fatigued toward COVID-19 relevant topics. This finding was consistent with past studies which discovered the relationship between media usage and IO in different settings. For instance, one study demonstrated that American newsreaders were more likely to feel overwhelmed toward the news content if their preferred news outlet was Facebook (45). Besides, during the earlier stage of the COVID-19 outbreak in South Korea, Hong and Kim (20) found that Koreans were more likely to suffer from IO when they consumed more COVID-19 information from online news sites. Therefore, our results double-confirmed this proposition tradition regarding the relationship between media consumption and IO.

Apart from social media engagement, our model also included interpersonal communication as another health information behavior because it has been identified as a vital information channel during an infectious disease outbreak (2, 46). Surprisingly, our results revealed that interpersonal communication was adversely associated with IO. Meaning, when someone discusses more COVID-19 topics with their family members and friends, the likelihood of feeling overwhelmed toward COVID-19 information reduced. Unlike another finding that reported positive relationship between information engagements on social media and IO, this result's direction diverges from our expectation. In addition, the negative relationship between interpersonal communication and IO was inconsistent with Hong and Kim (20), which suggests that interpersonal communication was positively associated with IO in the context of the COVID-19 in South Korea. To explain this inconsistency, some reasons are important to note. First, the process of communicating health topics with interpersonal networks involves real-time interaction, which allows individuals to express their ideas, comment on others' statements, and receive feedback concurrently. Unlike social media, which always directs individuals to the most relevant information based on specific algorithms, resulting in a sea of information that may or may not be of interest to individuals, bidirectional or multidimensional face-to-face communication allows an individual to decide what health topics he or she is interested in, which reduces uncertainty and anxiety about the health threat and thus promotes health outcomes (30, 47). Second, in terms of theoretical evidence, interpersonal communication has been conceptualized as one type of metacognitive processing strategy in health knowledge acquisition theories, especially the Cognitive Mediation Model (CMM) (2, 31). Individuals usually talk about the information they learned from media consumption with family and friends, which helps enhance their knowledge structure. As discussing and sharing COVID-19 information with interpersonal networks aids in information digestion, the negative association between interpersonal communication and IO was, therefore, reasonable.

IO was the consequence of two health information behaviors in the conceptual model, which served as the proximal outcome. We next examined whether IO predicts further consequences, such as a long-term mental health condition—perceived stress. The result supported this hypothesis: IO positively predicted perceived stress. When people are overwhelmed with COVID-19 information, they were more likely to suffer from long-term stress. This finding explained how the negative consequences of health information engagement might lead to more mental health issues, extending the scholarship pertaining to the way extant studies understand IO and its relationship with mental health symptoms. For example, one study conducted during the earlier stages of the COVID-19 outbreak in China solely examined the association between IO and anxiety and cognitive dissonance but ignored how IO is triggered (19). Similarly, another study in the UK also analyzed the relationship between COVID-19 IO and fear and fatigue of using social media while neglecting the mechanism of triggering IO (10). To further analyze the role of IO as a proximal outcome from health information behaviors to a mental health state, we performed mediation analysis, and the results confirmed that IO mediated the association between health information behaviors and long-term stress. Hence, our findings proffered conceptual guidance to researchers to better understand this pathway, from the causes of IO to the mental health effects.

Regarding risk perception, which we included it as the moderator in our conceptual model, although only one moderated mediation pathway demonstrated a significant effect, it is still noteworthy. Based on the results, the two-way interaction effect of risk perception and social media engagement had a negative relationship on the amount of IO. In other words, risk perception weakened the positive association between social media engagement and IO. For individuals who believed they had a higher chance of contracting the virus, the pandemic is severe to themselves and their community members; they were less likely to suffer from IO even if they encountered more COVID-19 information through social media usage. This result is in line with the proposition in Zhuang and Guan (34). When someone believes they are vulnerable to a health problem, their information-seeking experiences are more likely to prompt preventative behaviors, such as cancer screening. Meanwhile, our results detected a significant conditional indirect effect in the indirect pathway from social media engagement to perceived stress, mediated by IO, and this pathway was further moderated by COVID-19 risk perception. The negative effects of social media engagement on mental health conditions (i.e., stress) *via* the mediator of IO were stronger when individuals held a lower level of COVID-19 risk perception. This finding further highlights the powerful role of risk perception as a contextual factor in health information processing, which is very likely to influence immediate outcomes and further consequences, such as health conditions and attitudes toward health behaviors. This finding in general is further supported by the joint statement in studies based on the Health Belief Model (HBM) (48). Health beliefs such as perceived threat, efficacy, and potential benefits regarding prevention strategies mobilize healthy behaviors and reduce the likelihood of engaging with risky behaviors (49, 50). Therefore, linking our result with the statement in HBM research, we conclude that existing health beliefs influence health information behaviors and outcomes, especially through simple moderation and moderated mediation pathways.

## Implications and limitations

This study offers several implications. At the theoretical level, first, the proposed pathways in the conceptual model reflect the underpinning roles of IO in health information processing, especially during the COVID-19 infodemic. IO is the consequence of health information behaviors on social media and the negative metacognitive processing strategy, which causes an adverse health outcome (i.e., mental health condition). This presents opportunities for future studies to further investigate the functions and mechanisms of IO in health information management.

Second, we uncovered an essential personal health belief, namely, COVID-19 risk perception, as a moderator to understand the role of a contextual factor in the pathway from information behaviors to health outcome, a mental health condition. Even though only one moderated mediation effect was statistically significant, it still echoes and extends the scholarship in initial theoretical foundations, such as the CMM (31, 32) and the three-stage model of health promotion using interactive media (51).

Third, instead of following the traditional seeking and scanning approach to examine health information behaviors (52), we argued that due to the advancement of information technologies and the infodemic nature, it is hard to say whether individuals intentionally seek or unintentionally scan health information on social media. Thus, we reconsidered and simplified the measurement of health information behaviors on social media, only highlighting individuals' actions with COVID-19 information (i.e., information receiving and expressing) (36). It breaks new ground for future research regarding how online health information behaviors should be measured.

With regards to practical implications, first, our result revealed that during the infodemic era, receiving and expressing COVID-19 information on social media would trigger the chance of suffering from IO, which increases the stress level. As such, during the COVID-19 infodemic, social media companies and media practitioners should devote more efforts to censor and manage relevant content on their platforms, especially those from opinion leaders, online influencers, and other public accounts that have numerous followers. It can create and maintain a less-conflicted information environment for the users, where they then can obtain necessary health knowledge instead of causing IO and other negative health outcomes. Second, we found that COVID-19 risk perception as one type of personal health belief weakens the association between social media engagement and IO. Thus, governments, medical institutions, and health communicators should educate the public to be aware of the severity of the virus by strengthening their health beliefs. It can be done through both online and offline health promotion campaigns. Third, as interpersonal communication was negatively associated with IO in our conceptual model, it can be considered a powerful mechanism to decrease the chance of feeling IO and coping with mental health conditions. Health educators and campaign designers should highlight the crucial roles of face-to-face family communication and peer interaction in the infodemic era. Individuals are encouraged to discuss COVID-19 topics and share their opinions with their social networks to reduce stress.

Despite the implications discussed above, there are some limitations in our study. First, since this study was conducted during a full lockdown period in Malaysia, we could only use a cross-sectional online survey with convenience sampling to recruit respondents. The sample selection contained bias. It failed to reflect the accurate demographic structure in Malaysia, especially the distribution of age, ethnicity, religion, and education levels. Hence, the generalizability and representability of our results can be further improved. Second, our findings were relatively context-centered. The COVID-19 situation in Malaysia was severe during the time the survey was being conducted. It appears to be a plausible reason why the majority of surveyed respondents perceived higher levels of IO and felt stress (i.e., mean values skewed to strongly agree). If researchers replicate this study in other countries have successfully managed the pandemic, the public's daily life will return to normal; the proposed model may not be supported. Third, only risk perception as the personal health belief was considered a moderator, and a relatively weak interaction was found. This indicates that the moderating effect was not robust enough. However, other health beliefs, such as efficacy perception, might also be important in these pathways, and even moderating power could be more robust than risk perception. Fourth, the measurement of stress we utilized had several methodological concerns. The original operationalization of perceived stress that we adopted included five statements apropos of emotions (38). However, because the original items involved reverse-scored items (*n* = 3) which might cause confusion to respondents due to the possible effect of linguistic skills of respondents, the variance and reliability scores of the construct were affected. Although reserve-scored items are necessary to avoid response bias among respondents, this advice should be interpreted with caution because reports have revealed that reverse-scored items may be confusing to respondents, and that the opposite of a construct reverse-scored may be fundamentally different from the construct (53). Due to the low Cronbach's alpha achieved during the pilot test, we deleted these items and retained only two positive items for analysis. We also noticed that Ngien and Jiang's (38) conceptualization of stress is equivocal as they derived this measurement from the MHI-5 (54), which predominately measure mental health in general, rather than just stress. Therefore, future studies should refine this measurement proposed by Ngien and Jiang's (38) or consider other ways to measure stress for capturing a more holistic understanding. Finally, this study only included perceived stress as a mental health condition. While stress is a most common long-term feeling in the COVID-19 context, other long-term symptoms, such as anxiety and depression, should be taken into account in future research.

## Data availability statement

The raw data supporting the conclusions of this article will be made available by the authors, without undue reservation.

## Ethics statement

The studies involving human participants were reviewed and approved by the Ethics Committee for Research Involving Human Subjects (JKEUPM), Universiti Putra Malaysia [UPM/TNCPI/RMC/JKEUPM/1.4.18.2 (JKEUPM)]. The patients/participants provided their written informed consent to participate in this study.

## Author contributions

THZ organized the idea, proposed the conceptual model, conducted the literature search, developed the survey instrument, conducted the analyses, and drafted the manuscript. JST guided the statistical analyses. JST, MW, J-NK, J-SJ, PKC, and AMZA reviewed the manuscript. THZ, JST, and MW revised the manuscript. All authors have critically reviewed, provided intellectual input to the manuscript, and approved the final version of the manuscript.

## Funding

This study was funded by a grant from Universiti Putra Malaysia, Geran Putra IPM (GP-IPM/2019/9681300).

## Conflict of interest

The authors declare that the research was conducted in the absence of any commercial or financial relationships that could be construed as a potential conflict of interest.

## Publisher's note

All claims expressed in this article are solely those of the authors and do not necessarily represent those of their affiliated organizations, or those of the publisher, the editors and the reviewers. Any product that may be evaluated in this article, or claim that may be made by its manufacturer, is not guaranteed or endorsed by the publisher.
