# Supplementary material for: Pathway linking health information behaviors to mental health condition during the COVID-19 infodemic: A moderated mediation analysis
Source: Front Public Health. 2022 Aug 29;10:924331. doi: 10.3389/fpubh.2022.924331 (PMC9464915; doi:10.3389/fpubh.2022.924331)
Supplement: Supplementary file 1 [file Data_Sheet_1.PDF]

Survey Questionnaire (English)

SECTION 1 Social Media Engagement

		Not at all					*Very Frequently*
1	How often did you **receive** COVID-19 information on social media platforms e.g., Facebook, Twitter, Instagram, WhatsApp, WeChat, etc…) during the last **7 days**?	1	2	3	4	5	6
2	How often did you **express** COVID-19 information on social media platforms e.g., Facebook, Twitter, Instagram, WhatsApp, WeChat, etc…) during the last **7 days**?	1	2	3	4	5	6

SECTION 2 Interpersonal Communication

		Not at all					*Very Frequently*
1	How often do you discuss COVID-19 with your family members?	1	2	3	4	5	6
2	How often do you discuss COVID-19 with your friends?	1	2	3	4	5	6
3	How often do you discuss COVID-19 with your colleagues?	1	2	3	4	5	6
4	How often do you discuss COVID-19 with healthcare providers?	1	2	3	4	5	6

SECTION 3 Information Overload

		Strongly Disagree					*Strongly Agree*
1	There are so many different recommendations about preventing COVID-19, so it's hard to know which ones I should follow.	1	2	3	4	5	6
2	It has gotten to the point where I don't even care to hear new information about COVID-19.	1	2	3	4	5	6
3	Information about COVID-19 all starts to sound the same after a while.	1	2	3	4	5	6
4	I forget most COVID-19 information right after I hear it.	1	2	3	4	5	6
5	I feel overloaded by the amount of information about COVID-19 that I am supposed to know.	1	2	3	4	5	6

SECTION 4 Perceived Stress

		Strongly Disagree					*Strongly Agree*
1	Currently I feel so down in the dumps that nothing could cheer me up.	1	2	3	4	5	6
2	Currently I feel downhearted and blue.	1	2	3	4	5	6

SECTION 5 Risk Perception

		Strongly Disagree					*Strongly Agree*
1	The problem of COVID-19 outbreak is important to me.	1	2	3	4	5	6
2	I am worried that I will be infected with COVID-19 in the future.	1	2	3	4	5	6
3	It is likely that I will be infected with COVID-19.	1	2	3	4	5	6
4	I believe that I am at risk of being contracting with COVID-19.	1	2	3	4	5	6
5	The problem of COVID-19 outbreak is important to people in my country.	1	2	3	4	5	6
6	I am worried that people in my country will be infected with COVID-19 in the future.	1	2	3	4	5	6
7	The problem of COVID-19 outbreak is important to people living abroad.	1	2	3	4	5	6

SECTION 6 Social-Demographic Factors

1. What is your gender?

   (a) Male *(b) Female*

2. What is your Ethnicity?

   (a) Malay *(b) Chinese* *(C) Indian* *(D) Bumiputra* *(E) Others*

3. What is your religion?

   (a) Buddhism
   (b) Christianity
   (c) Islam
   (d) Taoism & Traditional Chinese Beliefs
   (e) Hinduism
   (f) None/Atheism
   (g) Others

4. What is your age? _____

5. What is the highest education level you completed?

   (a) No Formal Education
   (b) Primary School
   (c) Secondary School (SRP/PMR/PT3/MCE/SPM/SPM(V))
   (d) High School (HSC/STPM/ STAM)
   (e) Diploma

(f) Bachelor's Degree

(g) Postgraduate Degree

6. Where are you staying during COVID-19 outbreak?

(a) Metropolitan Area (i.e., Klang Valley)

(b) Urban Area

(c) Rural Area
